# Lung Protective Ventilation- Compliance With Best Practice Guidance: Critical Care Network Audit

**DOI:** 10.1186/2197-425X-3-S1-A508

**Published:** 2015-10-01

**Authors:** RM Gale, C Goddard, J Snell

**Affiliations:** Royal Liverpool University Hospital, Intensive Care, Liverpool, United Kingdom; Southport and Ormskirk NHS Trust, Anaesthesia and Intensive Care, Southport, United Kingdom; University Hospital Aintree, Intensive Care, Liverpool, United Kingdom

## Introduction

Since the ARDSNET study in 2000 [1],there has been a recognised best practice method of ventilation demonstrated to reduce mortality and morbidity in patients with acute lung injury. There is growing evidence that adopting a lower tidal volume strategy is beneficial for other patient groups [2].

## Objectives

To undertake a snapshot ventilation audit across Merseyside critical care units to evaluate regional compliance with best practice and to see if critical care network guidance is appropriate or required to improve performance.

## Methods

The audit design was a multi centre 2 day snapshot audit undertaken for 2 consecutive 24hr periods in July 2013. Data was collected on a standard proforma by nominated data collectors in each critical care unit. the audit standards were adopted from ARDSNET with a target tidal ventilation of 6ml/kg (based on ideal body weight, calculated from patients ulnar length), peak inspiratory pressure of 30cmH_2_0 and judicious use of PEEP. FiO2 data was also collected.

## Results

8 of 11 critical care units participated and were anonymised. Data was collected on 41 patients all of whom were either fully ventilated (CMV type mode) or receiving an assisted ventilatory mode (ASB type mode). Hours spent on CPAP based circuits were excluded. Patients were ventilated for a variety of reasons.

1317 hours were analysed, 885 hours were CMV and 432 hours were ASB. Every unit had mean tidal volumes >6ml/kg IBW for both CMV and ASB ventilation. 50% of units had a mean tidal volume of >8ml/kg. Only 8% of total CMV ventilated hours were < 6ml/kg. 5/8 units spent over 10% of their ventilated hours at tidal volumes of 9-12ml/kg. of the units with patients on ASB ventilation, compliance with TV < 6ml/kg varied from 0-45%.Only 4.2% of CMV ventilated hours were with PIP >30cmH_2_0. FiO2 was > 0.5 for 11% of ventilated hours and the level of PEEP used was very variable.

## Conclusions

The poor compliance was felt to be due to overestimation of patient weight. the results of the audit have been disseminated to each unit with the main recommendations of:

1. Each unit to introduce a rapid, easy method of calculating IBW.
2. Clear documentation of daily ventilation targets
3. Empower all multidisciplinary team members to respond to inappropriate ventilation.

The regional ventilator care bundle has been updated with an additional section for lung protective ventilation. the plan is for a repeat region wide audit now the ventilator care bundle and the audit results have been dispersed.

## Acknowledgment

Dr P Bamford, Dr N Hettiarachichi, Dr L Khoo, Dr L Ruff, Dr T Seddon, Dr K Tizard, Dr D Whitmore, Dr J Wong.

## References

[1] The Acute Respiratory Distress Syndrome Network (2000). Ventilation with lower tidal volumes as compared with traditional tidal volumes for Acute Lung Injury and Acute Respiratory Distress Syndrome. N Engl J Med.

[2] Gu WJ, Wang F, Lui JC (2015). Effect of lung protective ventilation with lower tidal volumes on clinical outcomes among patients undergoing surgery: a meta-analysis of randomised controlled trials. CMAJ.

